# Development of a Halochromic, Antimicrobial, and Antioxidant Starch-Based Film Containing Phenolic Extract from Jaboticaba Peel

**DOI:** 10.3390/foods12030653

**Published:** 2023-02-02

**Authors:** Rafaela F. Luz, Richard D. R. Ferreira, Cassio N. S. Silva, Bruna M. Miranda, Roberta H. Piccoli, Monique S. Silva, Ladyslene C. Paula, Maria Inês G. Leles, Kátia F. Fernandes, Maurício V. Cruz, Karla A. Batista

**Affiliations:** 1 Federal University of Goias, Samambaia Campus, Goiânia 74690-900, GO, Brazil; 2 Food Science Department, Federal University of Lavras, Lavras 37200-000, MG, Brazil; 3 Department of Food Engineering, Federal University of Rondônia, Ariquemes 76870-000, RO, Brazil; 4 Federal Institute for Education, Science, and Technology of Goias, Goiânia 74270-040, GO, Brazil

**Keywords:** bioactive film, active packaging, UV-absorber, phenolic extract

## Abstract

In this study, the antioxidant, antimicrobial, mechanical, optical, and barrier attributes of *Solanum lycocarpum* starch bio-based edible films incorporated with a phenolic extract from jaboticaba peel were investigated. Aiming to determine the effect of the polymers and the phenolic extract on the properties of the films, a three-factor simplex-lattice design was employed, and the formulation optimization was based on the produced films’ antioxidant potential. The optimized formulation of the starch-PEJP film showed a reddish-pink color with no cracks or bubbles and 91% antioxidant activity against DPPH radical. The optimized starch-PEJP film showed good transparency properties and a potent UV-blocking action, presenting color variation as a function of the pH values. The optimized film was also considerably resistant and highly flexible, showing a water vapor permeability of 3.28 × 10^−6^ g m^−1^ h^−1^ Pa^−1^. The microbial permeation test and antimicrobial evaluation demonstrated that the optimized starch-PEJP film avoided microbial contamination and was potent in reducing the growth of *Escherichia coli*, *Staphylococcus aureus*, and *Salmonella* spp. In summary, the active starch-PEJP film showed great potential as an environmentally friendly and halochromic material, presenting antioxidant and antimicrobial properties and high UV-protecting activity.

## 1. Introduction

The current world demand for improvement in food quality and safety and the concomitant increased concern about the increase in plastic waste production has attracted interest towards the development of eco-friendly packaging presenting low environmental charge in their whole life cycle [1,2]. Recently, native and modified starches from nonconventional sources have become a promising alternative to materials to produce environmentally friendly packaging materials and edible coatings. The main advantage of these starches is related to avoiding competition with the food production chain since they are not commonly used in human nutrition. They are also attractive due to their easy manipulation, low cost, biodegradability, and nontoxicity [3,4]. In the last decade, several studies have been developed aiming to evaluate the possible use of these nonconventional starch sources as food packaging able to: (i) avoid weight loss and oxidation [5,6]; (ii) improve the nutritional quality, sensory attributes, and shelf-life of perishable foods [5,7,8,9]; (iii) evaluate and monitor the freshness of food products [10,11]; and (iv) inhibit or reduce the microbial spoilage in different food matrices [1,2,12].

Amongst the nonconventional starch sources, *Solanum lycocarpum* fruit is an excellent source for starch obtention. *S. lycocarpum* is a common and abundant plant in the Brazilian Cerrado that presents a high resistance to hydric and climatic stress, surviving and fructifying along the year [13]. Its fruits can vary in size, weighing between 400 g and 900 g, and are not commonly used for human nutrition. This makes this plant a desirable target for starch isolation to develop eco-friendly materials, such as packaging or edible coatings and films.

Currently, starch films have been enriched with antioxidant, antimicrobial, and color change indicators to improve the active properties of these films, monitor the freshness, and prolong the shelf-life of perishable products [3,4,14]. Additionally, the possibility of developing innovative smart packaging to monitor the food condition supports the trends in sustainability by reducing food waste and losses in the food supply chain. However, many synthetic antioxidants, antimicrobials, and color indicators can harm human health or even pollutants. Therefore, natural additives are preferred over the former synthetic molecules concerning food safety and consumers’ health. Polyphenolic compounds stand out as powerful natural antioxidants due to their reducing properties useful in free radical scavenging. These compounds can also have UV light protection properties and antimicrobial action, which can delay oxidative processes and prevent or diminish microbial food spoilage [2,15].

Jaboticaba (*Plinia cauliflora*) is a Brazilian fruit with a purple peel that is considered a residue of the processing of wine and juice industries [11]. Among the various phenolic extracts reported in the literature, the extract of jaboticaba peel figures as a rich source of polyphenolic compounds with potent antioxidant and antimicrobial activity [9,10]. Thus, using jaboticaba peel as a source of phenolic compounds has the advantage of reutilizing an agro-industrial residue to develop low-cost and highly bioactive packaging materials or edible films.

Considering the complexity of optimizing film/coating formulations containing more than one ingredient, experimental mixture designs have been employed to reduce the time and costs associated with the film production process [16,17,18]. Amongst the most common mixture designs, the simplex-lattice design is a technique that allows obtaining a predictive mathematical representation of the relationship between mixture factors and responses with fewer observations, being possible to determine the optimal conditions for a formulation with high accuracy, quality, and low time and cost [19,20,21]. Compared to the conventional change of the one-factor-at-a-time approach, the use of mixture design has as the main advantage the possibility of evaluating not only the impact of the isolated variable on the response, but also the interactions between the components of the mixture [19].

Therefore, this study aimed to develop starch bio-based materials incorporated with a phenolic extract from jaboticaba peel (PEJP) as an active component. The film composition was optimized using a three-factor simplex-lattice mixture design. The optimized formulation was characterized by its morphological, physical, optical, mechanical, antioxidant, barrier, pH responsivity, and antimicrobial properties.

## 2. Materials and Methods

### 2.1. Materials

Citric acid (purity ≥ 99.5%, ACS reagent), glycerol (purity ≥ 99.5%, ACS reagent), nitric acid (purity 70%, ACS reagent), ethanol (purity 95%, ACS reagent), methanol (purity 99.8%, ACS reagent), and hydrochloric acid (purity 37%, ACS reagent) were purchased from Dinâmica Contemporânea Ltd.a. (Itaiatuba, SP, Brazil). Sodium chloride (purity ≥ 99%, ACS reagent), anhydrous calcium chloride (purity ≥ 97%, ACS reagent), and sodium hydroxide (pellets, purity ≥ 97%, ACS reagent) were purchased from Neon (Suzano, SP, Brazil). Potassium bromide (ref. 221864, FTIR grade), 2,2-diphenyl-1-picrylhydrazyl (ref. D9132), nylon membrane (ref. WHA7402002), qualitative filter paper (ref. WHA1001325), and cyanidin-3-glycoside chloride (ref. PHL89616) were purchased from Sigma Aldrich Chemical Co. (St. Louis, MO, USA). All other chemicals used were of analytical grade, obtained from accredited companies, and were used as received.

### 2.2. Starch Isolation

The *S. lycocarpum* unripe fruits were collected in Cristianópolis-GO, Brazil (17°11′58″ S and 48°42′12″ W). The isolation of the starch from the fruits was performed in a sequential procedure to obtain insoluble (IS) and soluble starch (SS), following the methodologies described by Pascoal et al. [13] and Rodrigues et al. [22]. Firstly, unripe *S. lycocarpum* fruits were washed, dehulled, and the seeds were removed. The pulp (20 g) was mixed with 0.8% citric acid solution (100 mL) and milled in a blender (model TE-631/4, Piracicaba, SP, Brazil) for 5 min. The resultant dispersion was filtered through a sieve of 500 μm, and the solid residue was washed twice with 50 mL of water. The filtrate was left to decant for 12 h at 4 °C, and the IS was separated by centrifugation at 4 °C and 4000× *g* for 10 min (Centrifuge model 5804R, Eppendorf, Hamburg, Germany).

The filtration residue was sequentially dispersed in 100 mL of water acidified with nitric acid (2 mol L^−1^) to pH 1.0. The dispersion was incubated at 85 °C (±5 °C) for 30 min, under stirring. Then, the mixture was cooled at room temperature and filtered using a nylon membrane. The starch was isolated by adding 200 mL of cold ethanol to the dispersion. The system was incubated at 4 °C for 12 h, and the precipitated starch was separated by centrifugation at 4 °C and 4000× *g* for 10 min (Centrifuge model 5804R, Eppendorf, Hamburg, Germany). The isolated starches (IS and SS) were dried at room temperature (25 ± 2 °C) and stored in airtight vials.

### 2.3. Phenolic Extraction from Jaboticaba Peel

PEJP was prepared according to Montes et al. [23], using an acidic ethanol solution. The ripe fruits were harvested at Jaboticabal Farm & Winery, located in Nova Fátima, Hidrolândia-GO, Brazil (16°55′32.35″ S and 49°21′39.76″ W). The collected fruits were washed and pulped using an electric pulper (Itametal, Bonina 0.25 DF, Brazil). The peels were manually separated from the seeds and milled (Tecnal blender model TE-631/4, Piracicaba, SP, Brazil) in a solution of acidic ethanol (acidified with 2.0 mol L^−1^ HCl to pH 4.0) for 2 min, then macerated for 9 h at 4 °C. After maceration, the dispersion was filtered using qualitative filter paper. The filtrate was stored in amber vials at 4 °C. The phenolic [11] and anthocyanin contents [12] were determined for the later incorporation of the extract in the starch films. Anthocyanin content was calculated as cyanidin-3-glycoside (C3G, MW = 449.2 g mol^−1^). The absorbance of the diluted sample (A) was calculated according to Equation (1):

A = ((A_520_ − A_700_)pH_1.0_) − (A_520_ − A_700_)pH_4.5_) (1)

where A_520_ is the sample absorption at 520 nm and A_700_ is the sample absorption at 700 nm (UV-Vis Spectrophotometer Model 700PLUS, Femto, São Paulo, SP, Brazil).

The total anthocyanin content of the sample was calculated using Equation (2):

Anthocyanin (C3G) = (A . MW . DF . 1000)/(ε . MP)(2)

where A is de absorbance of the diluted sample (obtained from Equation (1)), MW is the molecular weight of the cyanidin-3-glycoside (C3G), DF is the dilution factor, ε is the coefficient of molar extinction of the C3G (26,900 L mol^−1^ cm^−1^), and MP is the molar pathway (1 cm).

### 2.4. Preparation of the Starch-PEJP Films

The starch-based films were produced as described by Prates and Ascheri [24], varying the content of polymers and PEJP. A three-factor simplex-lattice design augmented with interior points and overall centroid was used to evaluate the influence of the type of starch and PEJP on the antioxidant potential of starch-PEJP films (Table 1). The design was generated using the software Statistica 10.0 (Statsoft Inc., Tulsa, OK, USA, 1997).

For the film production, SS or IS was firstly dispersed in distilled water and autoclaved (Phoenix Luferco, model AV 50/2, Araraquara, SP, Brazil) at 121 °C for 20 min (1 Kgf cm^−2^). After autoclaving, the solution was cooled at room temperature (25 °C), and the required content of PEJP was added to the system. Glycerol (0.5% (*w*/*v*)) was added to all formulations as a plasticizing agent. The filmogenic dispersion was homogenized under magnetic stirring for 10 min, gently poured on acrylic molds, and left to dry at room temperature until complete solvent evaporation. The dried films were detached from the molds and stored in plastic vials for further analysis.

### 2.5. Characterization of the Starch-PEJP Films

#### 2.5.1. Antioxidant Activity against DPPH Radical

The antioxidant activity of the starch-PEJP films was determined using an adapted methodology from Brand-Williams et al. [25]. Samples of 2 cm^2^ were immersed in 1.5 mL of DPPH (2,2-diphenyl-1-picrylhydrazyl) solution (0.15 mmol L^−1^ in 80% *v*/*v* methanol) and incubated for 60 min at room temperature (25 °C) under orbital stirring. The decrease in the absorbance of the resulting solution was monitored at 520 nm in a UV-Visible spectrophotometer (SP-220, Biospectro, São Paulo, BR). All measurements were performed in triplicates. The antioxidant activity was calculated as a percentage of DPPH radical scavenging using Equation (3):

DPPH scavenging (%) = ((A_s_ − A_a_)/A_s_) . 100

where A_s_ is the absorbance of the standard DPPH solution at 520 nm and A_a_ is the absorbance of the mixture reaction containing the tested samples at 520 nm.

#### 2.5.2. Scanning Electron Microscopy (SEM)

The surface and cross-sectional morphologies of the optimized starch-PEJP film were visualized using a JEOL JSM-IT300 scanning electron microscope (Jeol Ltd., Welwyn Garden City, Hertfordshire, UK). The samples were mounted on an aluminum stub using carbon-coated double-sided adhesive tape, sputter-coated with a layer of gold under vacuum (Coating system model DESK V, Denton Vacuum, Moorestown, NJ, USA), and examined using an accelerating voltage of 5 kV and a magnification varying from 500 to 2000 times. SEM experiments and analyses were performed in the Laboratório Multiusuário de Microscopia de Alta Resolução (LabMic) at the Federal University of Goias, GO, Brazil.

#### 2.5.3. Fourier Transform Infrared (FTIR) Spectroscopy

FTIR spectra of the isolated starches and the optimized starch-PEJP film were acquired on a PerkinElmer FTIR spectrometer (Spectrum 400, PerkinElmer, Inc., Waltham, MA, USA). For the test, samples were milled and mixed with potassium bromide (KBr), and the pellets were analyzed in the range of 4000–400 cm^−1^ (resolution of 2 cm^−1^), with 16 scans recorded [26].

#### 2.5.4. Mechanical Properties

The mechanical properties such as tensile strength, elongation at break, and Young’s Modulus of the optimized starch-PEJP films were measured according to ASTM D882-02 standard [27], using a Lloyd texture analyzer (model TA1, Lloyd/Ametek, Largo, FL, USA). Starch-PEJP films with uniform thickness were cut into 50 × 200 mm strips using a sharp stainless cutter, and tests were performed using an initial grip separation of 100 mm and a crosshead speed of 1 mm min^−1^. The thickness of the films was determined using a digital micrometer (Mitutoyo Corp., Tokyo, Japan) at 10 random positions in the sample.

#### 2.5.5. Thermal Properties

Thermal properties of the optimized starch-PEJP films were accessed by Thermogravimetric Analysis (TGA) using a TGA/DSC equipment (Mettler Toledo, Model 822^e^, Barueri, SP, Brazil). The analysis was conducted under a nitrogen atmosphere at a heating rate of 10 °C min^−1^. Samples of approximately 10 mg of the starch-PEJP film were cut as small pieces, placed into an aluminum pan, and heated from 30 °C to 500 °C. The TGA/DTG analysis was performed in the Laboratório de Métodos de Extração e Separação (LAMES) at the Federal University of Goias, GO, Brazil.

#### 2.5.6. Optical Properties

The light barrier properties of the starch-PEJP film against visible and ultraviolet light were determined by measuring the sample absorbance at a wavelength between 200 and 800 nm using a UV-Visible spectrophotometer (Model 700PLUS, Femto, São Paulo, SP, Brazil). Samples with uniform thickness were cut into 1 × 4 cm strips and placed in the spectrophotometer test cell, and the spectrum was recorded using air as a standard reference. For the opacity determination [28], the absorbance was checked at 600 nm, and the opacity of the starch-PEJP film was calculated using Equation (3):

Opacity (A.mm^−1^) = A_600_/X (3)

where, A_600_ is the absorbance value at 600 nm, and X is the thickness of the starch-PEJP film (mm).

For the transparency tests [29], the transmittance was checked at 600 nm, and the transparency of the films was determined using Equation (4):

Transparency (%.mm^−1^) = logT_600_/X (4)

where, T_600_ is the percentage of transmittance at 600 nm, and X is the film thickness (mm).

#### 2.5.7. Color Evaluation at Different pH Values

To evaluate the halochromic potential of the starch-PEJP film, 25 μL of different pH solutions were dropped into the film’s surface, and the color changes were registered after 15 min, using a digital camera (Samsung NX 3000). In the tests using acid pH it was used a 10 mmol·L^−1^ HCl solution for achieve pH = 2.0 and 0.1 mmol·L^−1^ HCL solution for pH 4.0. Milli-Q water was used as the standard for neutral pH (pH = 7.0) and 0.1 mmol·L^−1^ NaOH solution was the standard for alkaline pH (pH = 9.0).

#### 2.5.8. Water Solubility

To evaluate the water solubility of the starch-PEJP film, it was used an adapted methodology from Rodrigues et al. [30]. Starch-PEJP samples of 2 cm^2^ were dried at 105 °C for 24 h and weighed. The dried films were immersed in 30 mL of distilled water and stirred at room temperature for 24 h. The flasks containing the film were filtered using a qualitative filter paper (Whatman, WHA1001325, Sigma Aldrich), the residues were dried at 105 °C for 24 h, and their final weight was determined. The water solubility was expressed as percentage.

#### 2.5.9. Water Vapor Permeability (WVP)

The water vapor permeability (WVP) of the starch-PEJP films was measured gravimetrically according to the methodology described by Ghasemlou et al. [31]. The starch-PEJP films (discs of 5.4 cm^2^) were placed on the top of glass cups, filled with 25 g of anhydrous calcium chloride (0% RH), and the edges were covered with parafilm^®^ M to guarantee sealing. After this, each glass cup was placed in a desiccator maintained at 75% RH with a sodium-chloride-saturated solution (LabSynth, Diadema, São Paulo, Brazil) and stored at room temperature (25 °C ± 2 °C). The permeability of the starch-PEJP films was determined by linear regression of the constant mass transfer region between weight gain (g) and time (h) which was correlated with the exposed area to determine WVP, as described in Equation (5):

WVP (g m^−1^ h^−1^ Pa^−1^) = (Δm/A.Δt) . (X/Δp) (5)

where Δm/Δt is the slope of the weight-versus-time plot (g h^−1^), X is the average film thickness (m), A is the area of the exposed film surface (m^2^), and Δp is the water vapor pressure difference between the two sides of the film (1753.55 Pa). WVP was measured for four replicated samples.

#### 2.5.10. Antimicrobial Tests

The capacity to avoid microbial infiltration was tested according to the methodology described by Rodrigues et al. [30]. The starch-PEJP films were cut into discs of 1.5 cm^2^ (test area of 1.4 cm^2^), placed on the top of flasks containing 2 mL of nutrient Broth (NB, Kasvi, São José dos Pinhais, PR, Brazil), and covered the edges with parafilm^®^ M to guarantee sealing. The flasks were left in an open environment at room temperature (25 °C) for 10 days. Open flasks were used as a positive control, and flasks closed with a silicon stopper were used as a negative control. The occurrence of turbidity of the nutrient broth in any flask was recorded as microbial contamination.

The antimicrobial activity of the starch-PEJP films was evaluated using the agar diffusion method described by Agarwal et al. [32], with some modifications. Spread plates of BHI agar (Kasvi, São José dos Pinhais, Paraná, Brazil) were inoculated with 10 μL (10^8^ CFU mL^−1^) of different microorganisms (*Escherichia coli* EPEC CDC O55, *Staphylococcus aureus* ATCC 13565, and *Salmonella* spp. ATCC S64). Then, starch-PEJP films were cut into 4.0 × 1.0 cm strips and laid onto the inoculated plate’s surface. Next, the plate was incubated at 37 °C for 24 h. Antimicrobial efficiency was determined in four replicates by evaluating halo formation around the films and observing microbial growth density.

### 2.6. Statistical Analysis

The three-factor simplex-lattice design results were analyzed by regression analysis coupled to response surface methodology (RSM) using the software Statistica 10.0 (Statsoft Inc., Tulsa, OK, USA, 1997). All measurements were performed at least in triplicate. Results were expressed as mean ± standard deviation (X ± SD). The model was simplified by dropping terms that were not statistically significant (*p* > 0.05) by ANOVA. The effect size of each variable and interaction on the antioxidant activity of the produced film was represented using a Pareto chart. The Pareto chart aims to identify the vital few variables that strongly contribute to the response. Pareto rules say that 10% to 20% of the tested elements will determine 60% to 80% of the response. These variables are essential items of concern and must be carefully considered. The desirability function was performed, and the optimum formulation was set to maximal antioxidant activity.

## 3. Results and Discussion

### 3.1. Production of the Starch-PEJP Films

Before the production of the starch-PEJP films, PEJP was characterized regarding the content of phenolic compounds and anthocyanin, and the results evidenced a phenolic content of 110.4 (± 0.2) μg GAE mL^−1^ of PEJP and an anthocyanin concentration of 103.2 (± 1.2) μg mL^−1^. SS and IS were evaluated regarding their antioxidant activity, and results showed 25.2% of DPPH scavenging activity for IS and 78.2% for SS.

To determine the best proportion of *S. lycocarpum* starches (IS and SS) and PEJP to produce a film with maximum antioxidant activity, 10 formulations were predicted by a three-factor simplex-lattice design. The starch-PEJP films generally presented a regular and uniform appearance without cracks or bubbles. The results of the antioxidant activity of the prepared starch-PEJP films are shown in Table 1.

**Figure 1:**
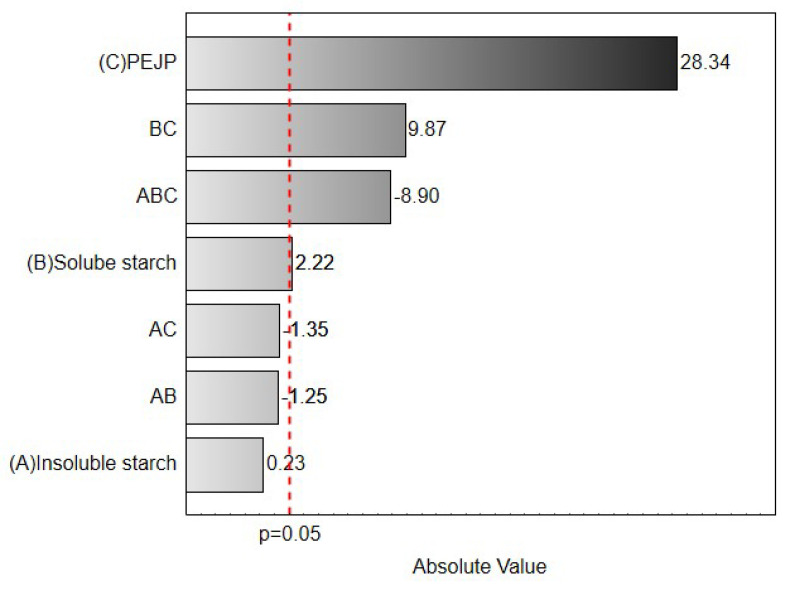
Pareto Chart showing the effect (absolute value) of the variables (SS, IS, and PEJP) on the antioxidant potential of the starch-PEJP films. The dashed line denotes the significance level −horizontal bars crossing the dashed line significantly interfere with the response.

As can be observed in Table 1, the type of starch and the content of PEJP significantly interfered with the antioxidant activity of the starch-PEJP films, being observed values from 0 to 86.93% of DPPH discoloration. Results from multivariate analysis evidenced that the most critical component that affected the films’ antioxidant potential was the proportion of PEJP (Figure 1). In general, the antioxidant activity of the films increased when increasing the proportion of PEJP. This was expected due to the high content of phenolics, especially anthocyanin molecules, observed in this extract [33].

**Figure 2:**
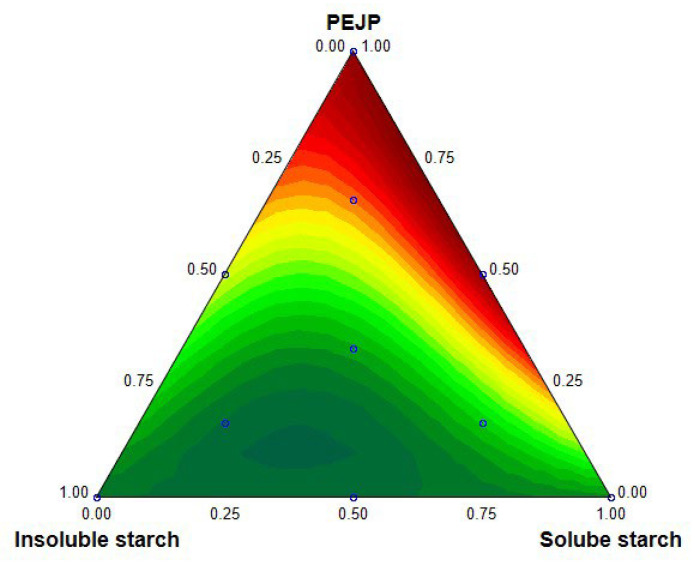
Two-dimensional response surface plot depicting the effect of the variables (SS, IS, and PEJP) on the antioxidant potential of the starch-PEJP films.

However, not only the content of PEJP contributed to the antioxidant potential of the starch-PEJP films, but also the content of SS and the combined effect of PEJP/SS improved the scavenging potential of the films against DPPH radical (Figure 2). The ability of phenolics to donate protons or produce stable intermediary radicals is the primary explanation for the improved antioxidant potential of PEJP [34]. On the other hand, the antioxidant activity showed by SS also contributed to the antioxidant potential evoked by the starch-PEJP films. The higher antioxidant activity of this starch may be due to the formation of saccharic acid residues in the anomeric regions of the starch as a result of the isolation process using nitric acid at high temperature.

### 3.2. Optimization of the Starch-PEJP Film Formulation

**Figure 3:**
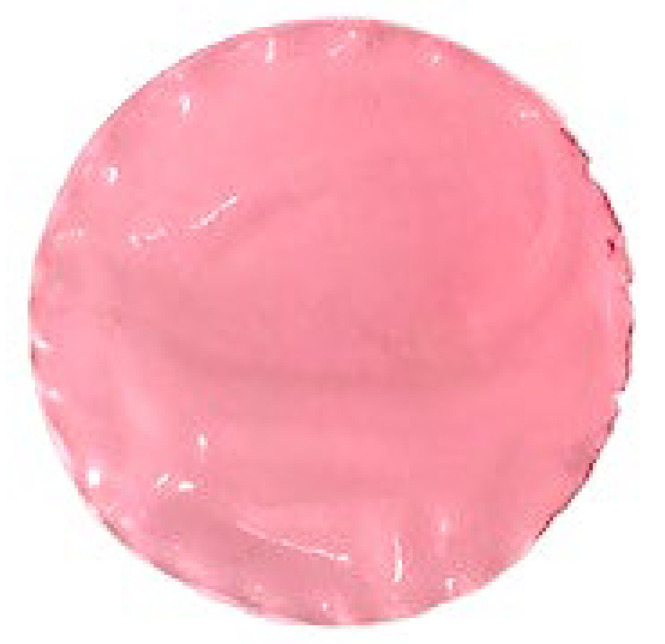
Macroscopic appearance of the optimized starch-PEJP film formulation.

The optimization of the formulation to achieve the maximum antioxidant activity was performed within the composition constraint using the desirability function. The optimal starch-PEJP composition predicted by the model included 1% IS, 0.87% SS and 375 μL of PEJP, with a projected antioxidant activity of 90.83% (d = 0.99). The experimental validation evidenced that the starch-PEJP film presented an antioxidant activity of 91.01% (±0.5), with a thickness of 81.8 μm (±0.6). In this optimized formulation, the starch-PEJP film showed a reddish-pink color, a regular and uniform appearance, and no cracks or bubbles (Figure 3).

### 3.3. Characterization of the Optimized Starch-PEJP Film

#### 3.3.1. Fourier Transform Infrared (FTIR) Spectroscopy

**Figure 4:**
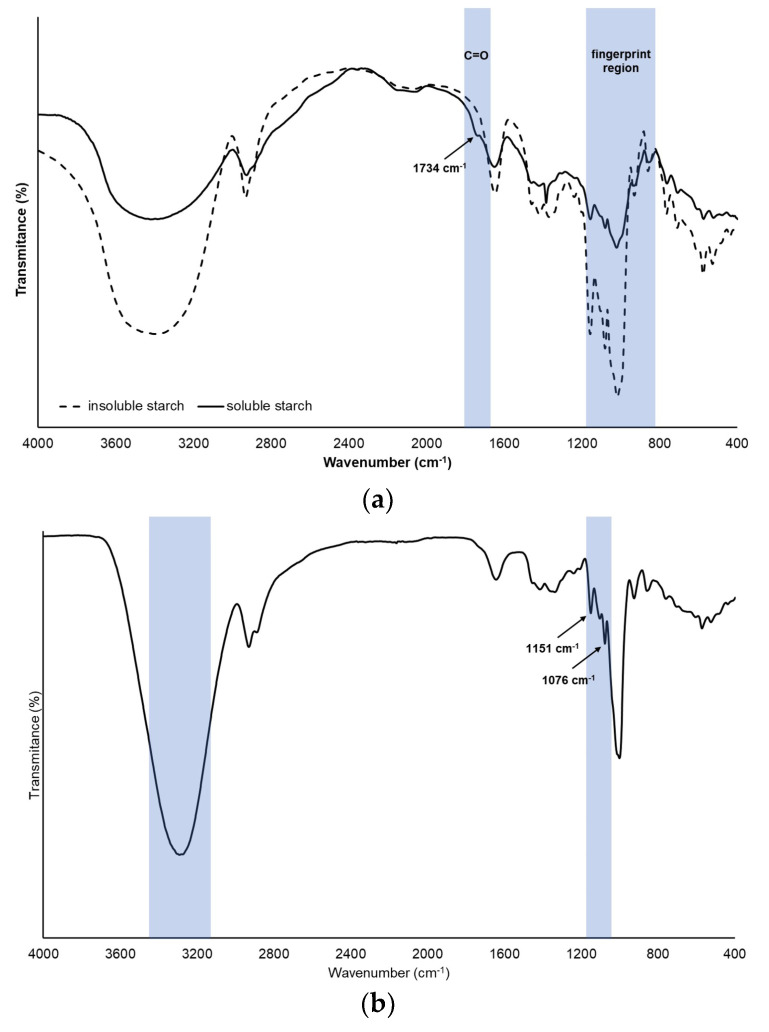
FTIR spectra from (**a**) soluble and insoluble starches isolated from *S. lycocarpum*; and (**b**) starch−PEJP films.

FTIR analysis was used to characterize the SS and IS and evaluate the interactions between the polymers and PEJP in the optimized formulation of the starch-PEJP film. The FTIR spectra of SS and IS (Figure 4a) showed typical bands of amylaceous materials, with the presence of a strong broad band in the region of 3600–300 cm^−2^ assigned to the stretching of -OH groups, a peak around 2940–2910 cm^−1^ related to the vibration of -CH bonds from glucose, and a peak in the region of 1647 cm^−1^ assigned to scissors vibrations of hydroxyl groups from hydration water. [13,35]. The chemical modification in the SS structure was evidenced in FTIR analysis (Figure 4a) by the presence of a band at 1734 cm^−1^, ascribed to the C=O stretching of carboxylic acid, which is absent in the FTIR spectrum of IS. However, the formation of saccharic acid in the anomeric regions of the starch molecule did not impair the backbone structure of the starch molecule, with typical peaks being observed in the region of 1200–800 cm^−1^ from both starches, SS and IS. This region is referred to as a fingerprint region for polysaccharides, presenting bands related to the stretching vibrations of C-O-C and C-O-H from glycosidic linkages [13,36].

From the FTIR spectrum of the optimized formulation for the starch-PEJP films (Figure 4b), it is possible to suggest the occurrence of an interaction at the molecular level between IS and SS, evidenced by the changes in the band at 3400 cm^−1^ observed in the native starches (Figure 4a), which became sharper and shifted to a lower wavenumber (3287 cm^−1^) in the produced film (Figure 4b). These alterations indicate that besides the presence of a different content of entrapped water, there is also an enhancement of the hydrogen interactions between the starch polymers, phenolic compounds, and even glycerol (as a plasticizer) [36,37,38]. In addition, the bands observed at 1151 cm^−1^ and 1076 cm^−1^ (Figure 4b) confirm the presence of phenolic compounds from PEJP, mainly anthocyanins such as cyanidin and delphinidin [33].

#### 3.3.2. Scanning Electron Microscopy

**Figure 5:**
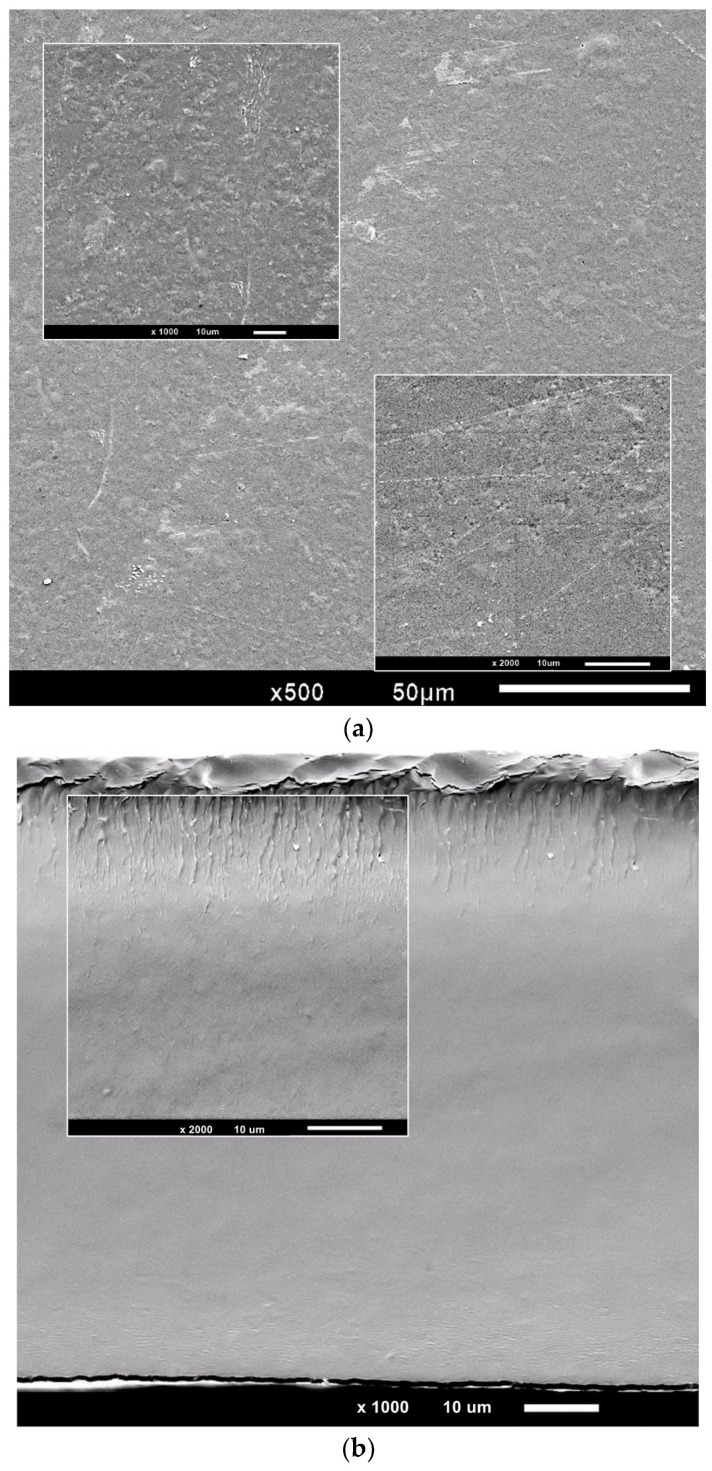
SEM micrographs of the (**a**) surface (**b**) cross-sectional morphology of the starch-PEJP film.

SEM analysis was used to evaluate the surface and cross-sectional morphology of the optimized formulation of the starch-PEJP film (Figure 5).

The produced film presented, in general, a homogeneous surface (Figure 5a), with some irregularities associated with the drying stress, which may be caused by the stress induced in the polymer chains upon solvent evaporation. On the other hand, the cross-sectional analysis of the starch-PEJP coating (Figure 5b) evidenced a compact, soft and uniform structure without vesicles or pores. These results suggest that PEJP was compatible with the other polymer components (IS and SS) and plasticizer (glycerol), resulting in a cohesive and stable three-dimensional matrix.

#### 3.3.3. Optical Properties

Despite the reddish-pink color observed in the optimized starch-PEJP film, it presented good transparency to visible light (Figure 3, Table 2) with a low opacity value in the visible spectrum (Table 2). High transparency values are associated with great homogeneity of the interactions between the compounds that constitute the polymer matrix, which was already confirmed by the structural analysis through SEM (Figure 5). In addition, the opacity of matrices used as food coating can contribute to avoiding food deterioration caused by exposure to visible and ultraviolet light, protecting the food against nutrient losses, discoloration, and development of off-flavors [5,29,39].

The presence of opacity in the starch-PEJP film may be an interesting property if considering its use as a packaging for oxidizable foods. Similar values of transparency and opacity were reported in other starch films and coatings containing phenolic extracts (Table 2). This optical behavior could be attributed to the selective light absorption of the polyphenols found in these extracts. However, not only the opacity at visible light is mandatory, but also it is important to determine the light absorption profile of a food package in the ultraviolet region (200–400 nm) since one of the desired characteristics of covering materials is that it should protect food from the effects of ultraviolet radiation.

**Figure 6:**
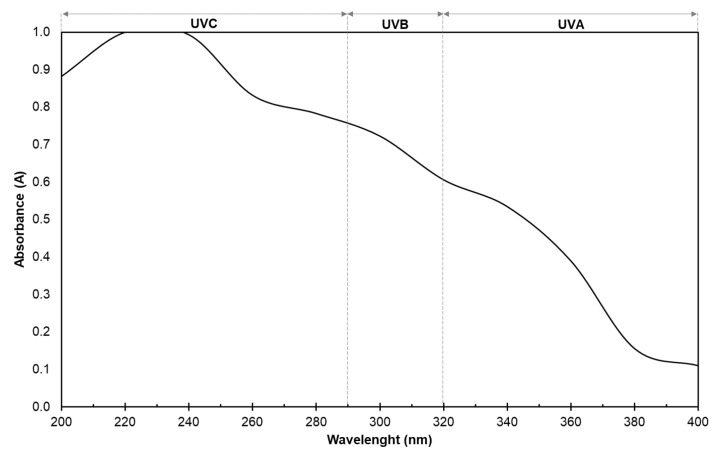
Ultraviolet absorption spectrum of the starch-PEJP film.

The results from the UV-blocking action of the starch-PEJP film showed a high absorption in the UV region (Figure 6), especially in the regions of UVC (200–290 nm) and UVB (290–320 nm). These results can be explained due to the chemical structures of the phenolic compounds found in PEJP, with the absorption profile being dependent on the organization of the aromatic rings and positions of the hydroxyl groups in the phenolic molecules [30]. Together with the already-mentioned antioxidant activity and the presence of visible opacity, the high barrier to UV radiation showed by the starch-PEJP film makes it a promising material to be used as a food covering since it contributes to the reduction in the oxidative processes and, therefore, can improve or extend the shelf-life of food, especially those oxidizable food products [3].

#### 3.3.4. Water Solubility

The water solubility of the optimized starch-PEJP film is shown in Table 2. Water solubility is an essential parameter to be evaluated for materials used as food packaging and covering since their resistance and integrity when in contact with water will determine their final applicability as packaging or coating [26,44]. The desired value for the solubility of a film will depend on its application or intended use. Hence, according to the results, the starch-PEJP matrix can act as an edible coating or film package for foods and foodstuffs with lower moisture content, with a special focus on oxidizable/perishable products.

#### 3.3.5. Water Vapor Permeability (WVP)

The water vapor permeability is a key characteristic to measure the quality of food packaging, enabling the determination the content of vapor molecules of water passing through the film. The WVP values of starch-biobased films can widely vary depending on the content of amylose and amylopectin of the starch used, the presence of chemical modifications, and complexation with other polymers and/or plasticizers [15,48,51]. The WVP of the starch-PEJP film is shown in Table 2, and the high WVP obtained is a result of the film microstructure and composition. Despite the homogeneous microstructure of the starch-PEJP film (Figure 5), the hydrophilic nature of the film network polymers may have contributed to obtaining a less crystalline material with increased free volume and molecular mobility, which favors the water movement between the film and the surrounding environment.

Similar studies (Table 2) also confirmed that the WVP behavior of starch-based films could be highly affected by the chemical properties of the starch and non-starch components used in the formulation. Aaliya et al. [2] and Wang et al. [15] have reported that a higher number of free hydroxyls and free glycerol in the film matrix can reduce the moisture barrier of starch-based films. In addition, the maintenance of a crystalline structure in the film matrix contributes to lowering the WVP values since the reduction in free volume and molecular mobility of the film network contributes to increasing the water resistance [36].

#### 3.3.6. Mechanical Properties

The mechanical properties of the optimized starch-PEJP film are shown in Table 2. Results from tensile strength (16.3 MPa) were higher than for other starch-based films and coatings, indicating that the starch-PEJP matrix is more resistant to tearing. Furthermore, the starch-PEJP film also presented a higher elongation at break (79.7%) than other starch bio-based films (Table 2). The elongation at break is a parameter that indicates the plastic behavior of the material. The high flexibility of the produced starch-PEJP film can be explained by the combined effect of glycerol and the phenolic compounds acting together as plasticizing agents, reducing the intermolecular forces between adjacent polymer chains as well as increasing the mobility of polymer chains on the film matrix, thus making the starch-PEJP film more extensible. The flexibility of the produced film was also confirmed by the determination of Young’s modulus (5.5 MPa). Young’s modulus is an essential parameter for measuring the stiffness of polymeric films, being observed a wide variation in this value as a function of the film composition (Table 2). In general, the higher Young’s modulus of the material, the stiffer this material is, with lower values indicating a film with elastic properties. For the starch-PEJP film, the low value of Young’s modulus (Table 2) suggested that the starch-PEJP is a soft material with a highly flexible matrix.

#### 3.3.7. Thermal Properties

**Figure 7:**
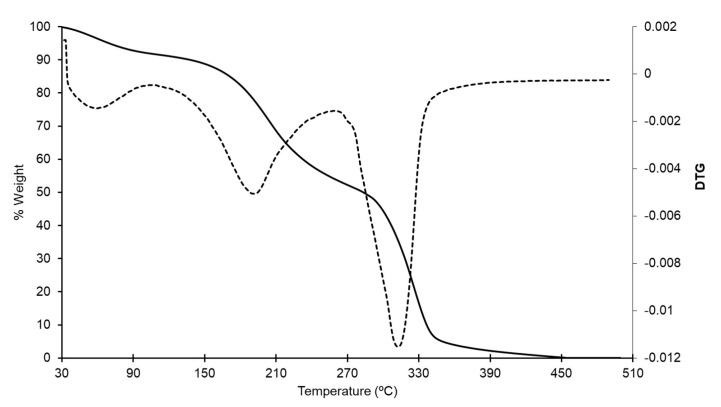
TGA and DTG thermograms of the starch-PEJP film.

The thermal stability of the starch-PEJP film was evaluated by TGA analysis. Figure 7 shows the TGA and DTG thermograms of the starch-PEJP film, where it is possible to observe three stages of degradation. In the first stage (70–100 °C), the descending trend of the TGA curve is relatively gentle, and the mass loss observed (8.5%) could be attributed to the volatilization of absorbed bounded water in the film and small molecules from the polymeric matrix [21]. The second stage occurs between 160 and 270 °C (peak at 200 °C), corresponding to the decomposition of organic materials of low molecular weight, such as glycerol and phenolic compounds. Additionally, at this temperature range, starch depolymerization and internal reorganization can occur, leading to the glass transition phenomenon, in which the polymer matrix changes from a hard to a soft feature [5].

The sharp descending curve of the third weight loss stage (280–330 °C) indicates the extensive degradation of the sample, with a weight loss of 48.9% being observed (Figure 7). In this stage the total decomposition of the material occurs due to the disruption of adjacent hydroxyl and glycosidic bonds between the polymers in the starch-PEJP film matrix, with the oxidation and complete decomposition of organic matter [5]. Above 450 °C, an evident stabilization of the TGA/DTG was observed, which was attributed to the endothermic crystallization that leads to carbonization and ash formation [21]. In general, TGA results indicated that the starch-PEJP film could withstand high temperatures, suggesting an organized and stable film matrix.

#### 3.3.8. Color Evaluation at Different pH Conditions

**Figure 8:**
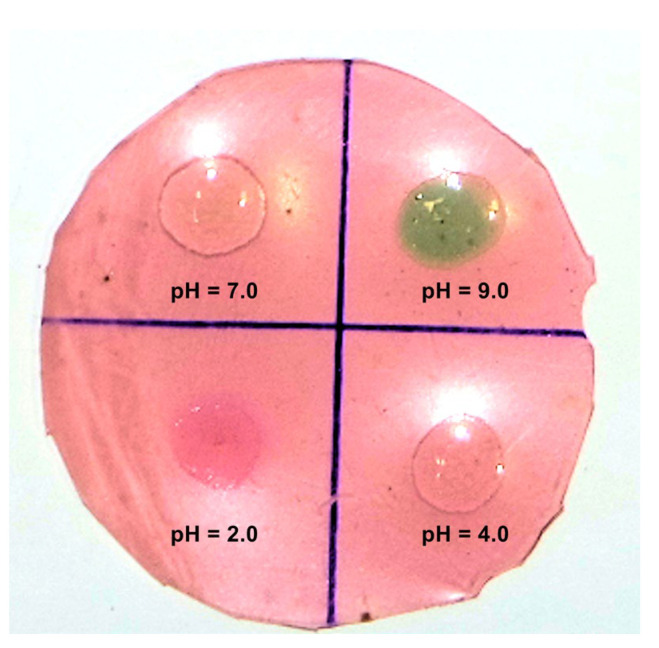
Color variation of the starch-PEJP film submitted to different pH conditions: photograph after 15 min of dropping the pH solution.

The color changing of starch-PEJP film at different pH values is shown in Figure 8. The color variation is associated with anthocyanins found in the PEJP [33]. The more reddish-pink color observed at acidic pH can be attributed to the conversion of the anthocyanin molecules to flavylium cations. The positive charge on the flavylium ion is delocalized over the heterocyclic part of the molecule, and this delocalization is responsible for the intensification of the reddish-pink color of the optimized starch-PEJP film [52,53].

At pH 4.0, the optimized starch-PEJP film did not present color alteration, and at pH 6.0, it was possible to observe a slight brownish tone starting to appear. This color change can be ascribed to the conjugation of water molecules to the heterocycle structure of the anthocyanin molecules and the increase in pH, which leads to a neutralization of the positive charges and the formation of the colorless carbinol pseudo-base [7,54]. When the pH value reached 9.0, the color of the starch-PEJP film changed from a reddish-pink to a greenish tone, an event probably associated with the transformation of anthocyanins into colored quinones and the methylation reaction on its aromatic ring [55].

In recent years, there has been growing interest in developing intelligent food packaging to reduce food waste and enhance consumer safety. The possibility of developing a smart coating of high sensitivity and safety that provides direct information without damaging the package integrity just by optical/visual color changes is of great interest [11,44]. Therefore, the ability of the starch-PEJP film to change its color as a function of pH variation allows its use as a natural indicator of the quality of foodstuffs with pH response of reactions of deterioration or also acts in real-time monitoring of food freshness. Furthermore, the straightforward interpretation of the response upon color change and the simplicity in function and operation are advantages of this system using a natural indicator compared to the commonly used electronic, electrochemical and enzymatic indicators [56].

#### 3.3.9. Antimicrobial Permeation

**Figure 9:**
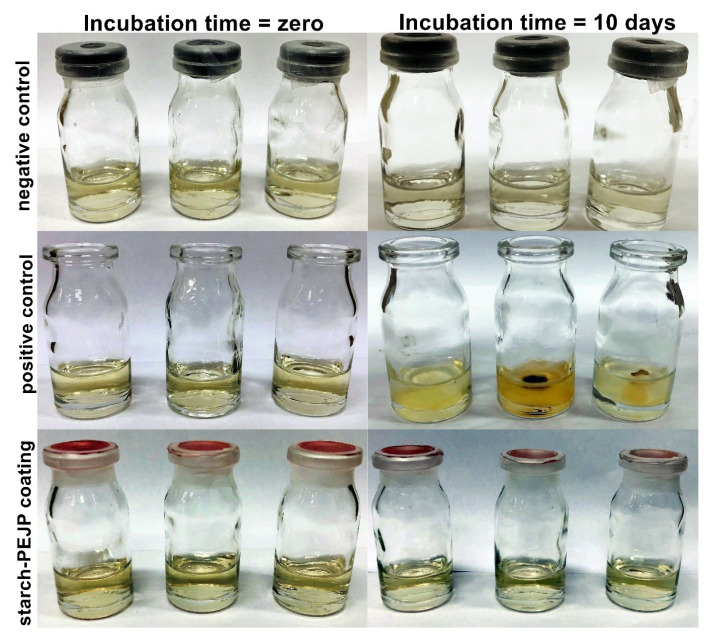
Photographs of antimicrobial permeation test before and after ten days of incubation at room temperature (25.0 ± 2.0 °C).

Aiming to evaluate the ability of the optimized starch-PEJP film to avoid microbial contamination and permeation, the microbial penetration was assessed, and the results are shown in Figure 9. Microorganisms that could penetrate through the film matrix would cause turbidity in the medium as a function of microbial growth. As can be observed in Figure 9, the positive control test did not present any restriction to the penetration of microorganisms from the environment to access the culture medium. Therefore, all flasks showed microbial contamination after ten days of storage under environmental conditions.

Considering the flasks covered with the starch-PEJP film, the results evidenced that the coating could prevent the penetration of microorganisms into the medium, being observed no visible microbial growth, and the maintenance of a transparent medium (Figure 9). This result can be due to two reasons: (1) the starch-PEJP film is a cohesive matrix, providing a physical barrier against microbial penetration; and (2) the intrinsic antimicrobial effect of the phenolic compounds from PEJP could also contribute to avoiding the microbial permeation. This is a very encouraging result for developing smart antimicrobial films with improved mechanical properties through the combination of starch with other bioactive compounds, which can be used as a food coating or packaging, reducing the primary contamination and increasing the shelf-life of the product.

#### 3.3.10. Antimicrobial Efficiency Assessment

**Figure 10:**
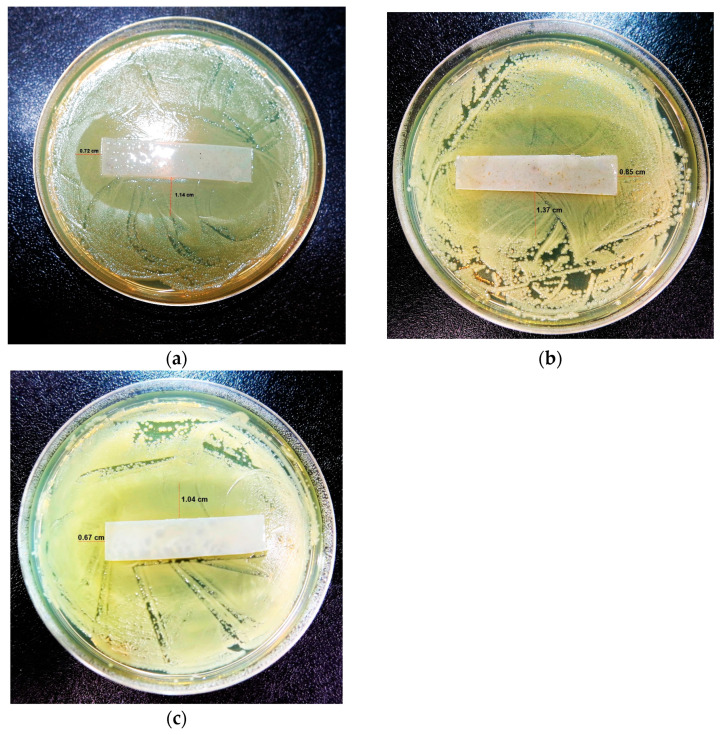
Antimicrobial efficacy tests of the starch-PEJP film against (**a**) *Staphylococcus aureus*; (**b**) *Escherichia coli*; and (**c**) *Salmonella* spp.

Together with avoiding microbial permeation, the inhibition of microbial growth is a desired feature for antimicrobial food packaging and coating materials [15]. To determine the antimicrobial efficiency of the optimized starch-PEJP film, we evaluated the size of the inhibitory region of food-derived bacteria, and the results are shown in Figure 10. As can be observed, the films were more effective towards the Gram-positive bacterium *Staphylococcus aureus* (Figure 10a) rather than the Gram-negative bacteria *Escherichia coli* (Figure 10b) and *Salmonella* spp. (Figure 10c). This antimicrobial behavior can be ascribed to the intrinsic tolerance of these different groups of microorganisms and the sensitivity of the molecules found in the PEJP.

The tolerance against antimicrobial molecules is strongly correlated with the structural differences between these two types of bacteria. It is possible that the absence of a lipid membrane in the cell wall of the Gram-positive bacteria could facilitate PEJP to enter and interfere with cellular metabolism. Nevertheless, the more complex nature of the cell wall of the Gram-negative strains, with a thick outer lipopolysaccharide membrane, makes difficult for the PEJP to pass through the outer layer and cause alterations in their cytoplasm [12,57].

Moreover, this partial inhibition can be ascribed to the concentration of PEJP used in the film production, which could be in insufficient quantity to completely inhibit the microbial load present in the plate. However, considering that it is possible to increase the content of PEJP by increasing the volume of film-forming solution used in the casting procedure, it is possible to infer that the starch-PEJP film is promising as a bioactive material containing natural antimicrobial compounds that avoid microbial penetration and diminish food microbial spoilage.

## 4. Conclusions

We obtained a halochromic starch-PEJP film with efficient antioxidant and antimicrobial activity in this study. The simplex-lattice mixture design was effective in obtaining mathematical models to predict response values. Consequently, it was a helpful tool to optimize the starch-PEJP formulation to the maximum antioxidant activity. The optimized formulation showed a reddish-pink to greenish color as a function of the pH, suggesting this film is an effective smart material to be used as a natural indicator for the quality of foodstuffs or even be used in the real-time monitoring of food freshness. Future studies are required to explore those applications.

The optimized starch-PEJP film also showed high extensibility with low stiffness and good properties of transparency and opacity. Furthermore, the starch-PEJP film demonstrated strong antimicrobial effectiveness, being an efficient barrier against microbial permeation and can reduce the growth of Gram-positive and Gram-negative bacteria. Therefore, the starch-PEJP film has potential applications in the food industry as bioactive coverage with active functionality as a natural indicator with potent antioxidant and antimicrobial properties. Additionally, the results of this study could be used as a guideline for producing environmentally friendly smart active films for the packaging of foods by simultaneously studying various variables and their effects on the material properties and bioactivities.

## Figures and Tables

**Table 1:** Mixture design and DPPH scavenging activity for the different formulations of starch-PEJP films.

Tests	Film Components ^1^			Antioxidant Activity (% DPPH Scavenging) ^2^
	SS (%)	IS (%)	PEJP (μL)	
E1	5.0	0.5	0	0
E2	1	2	0	8.54
E3	1	0.5	500	86.93
E4	3	1.25	0	0
E5	3	0.5	250	37.99
E6	1	1.25	250	86.72
E7	2.3	0.95	85	0
E8	3.7	0.75	85	10.12
E9	1.7	1.5	335	54.78
E10	1.7	0.75	165	14.01

^1^ SS = soluble starch from *S. lycocarpum*; IS = insoluble starch from *S. lycocarpum*; PEJP = phenolic extract from jaboticaba peel. ^2^ Results are presented as the mean of three determinations.

**Table 2:** Properties of the starch-PEJP compared to other similar starch bio-based films and coatings.

Properties	Optimized Starch-PEJP Film ^1^	Starch Bio-Based Films and/or Coatings with Phenolics	References
Transparency (% mm^−1^)	75.1 ± 1.9	40–84.36	[39,40,41,42]
Opacity (A mm^−1^)	7.5 ± 0.2	0.5–6.23	[3,14,29,40,43]
Water solubility (%)	59.1 ± 0.8	4.2–66.3	[8,40,43,44,45]
Water vapor permeability (g m^−1^ h^−1^ Pa^−1^)	3.28 ± 0.04 × 10^−6^	9.8 × 10^−11^–3.4 × 10^−4^	[2,15,36,46,47,48]
Tensile strength (MPa)	16.3 ± 0.7	0.6–19.9	[8,14,41,49,50]
Elongation (%)	79.7 ± 3.1	0.6–70.4	[8,14,39,40,50]
Young’s modulus (MPa)	5.5 ± 0.9	1.23–43.4	[3,8,49,50]

^1^ Results were expressed as the mean of at least three determinations.

## Data Availability

The data supporting this study’s findings are available from the corresponding author upon reasonable request.
